# Amelanotic Melanoma Masquerading as a Granular Cell Lesion

**DOI:** 10.1155/2013/924573

**Published:** 2013-02-25

**Authors:** Deepak Pandiar, Shaini Basheer, P. M. Shameena, S. Sudha, Lakshmi J. Dhana

**Affiliations:** Department of Oral and Maxillofacial Pathology, Government Dental College, Kozhikode, Kerala 673008, India

## Abstract

Amelanotic melanoma (AM) presents a diagnostic challenge due to its wide clinical presentations, lack of pigmentation, and varied histological appearances. Immunohistochemistry plays a crucial role in the diagnosis of these lesions. Amelanotic melanoma of oral mucosa is an uncommon lesion. We report a case of a 50-year-old male patient with a growth on the anterior mandibular gingiva of seven-month duration. In the present case, histologically, the tumour resembled a granular cell lesion, which has not been reported previously in AM. Diagnosis was possible by a sequential panel of immunohistochemical markers, of which finally vimentin, S100, HMB45, and Melan-A were positive. The tumor was surgically excised, and postsurgical radiotherapy was given.

## 1. Introduction

Malignant melanomas most commonly occur on skin (over 90%) and less commonly on mucosal surfaces, that is, slightly over 1% [1]. It accounts for approximately 4% of all skin cancers, but it is responsible for 79% of all skin-cancer-related deaths [2]. These lesions are classically pigmented but amelanotic subtype is also well documented in the literature, which is often difficult to diagnose and requires microscopic evaluation aided by immunohistochemistry for correct diagnosis. Thrity-four cases of amelanotic melanomas (AM) have been reported on oral mucosa and only three on the mandibular mucosa. This is to the best of our knowledge of the fourth case. Although different histological subtypes of malignant melanoma are known to occur, PubMed/Medline search did not revealany previous report of granular cell variant of malignant melanoma. 

## 2. Case Report

**Figure 1:**
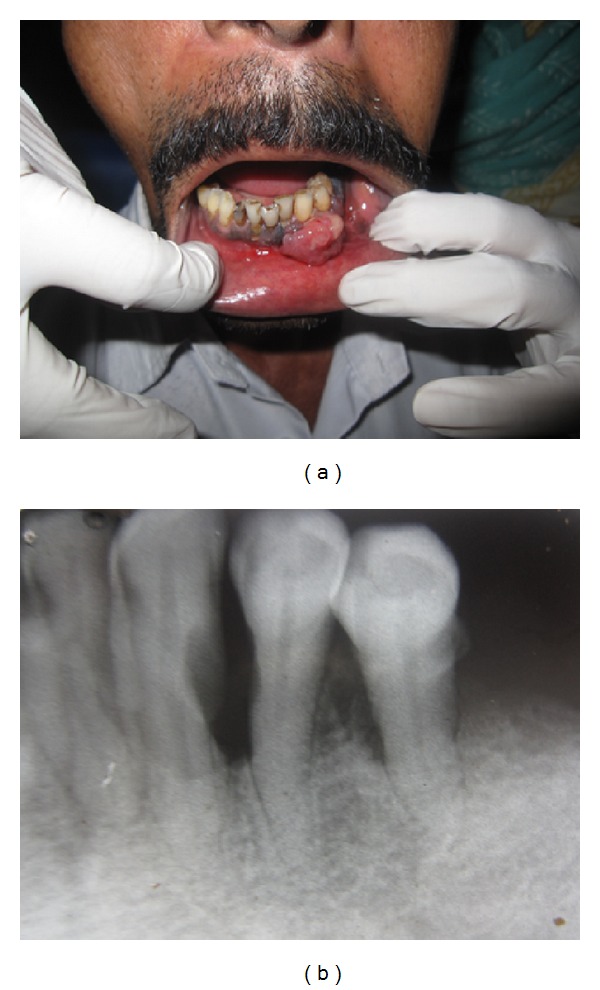
(a) Intraoral view of lesion in anterior mandibular region. (b) IOPA showing mild rarefaction and bone loss in the 33–35 region.

A 50-year-old male patient reported to the Department of Oral Pathology and Microbiology, Government. Dental College, Kozhikode, with a chief complaint of a growth on the lower anterior gingiva since 7 months. Medical history revealed that the patient was under medication for epilepsy and hypertension. He is a smoker (1 pack per day for the past 32 years) and occasionally takes alcohol. On examination, a growth of size about 3 × 2.5 × 1 cm was noticed on the labial gingiva extending from 31 to 34 region. The overlying mucosa was slightly erythematous and without any ulceration (Figure 1(a)). The lesion was sessile, nontender, and firm in consistency. There was no bleeding on palpation. Associated teeth were within normal limits. Intraoral periapical radiograph showed mild rarefaction of the alveolar bone in the 33–35 region (Figure 1(b)). Lymph nodes were not palpable. A provisional diagnosis of pyogenic granuloma was given. Incisional biopsy was done.

**Figure 2:**
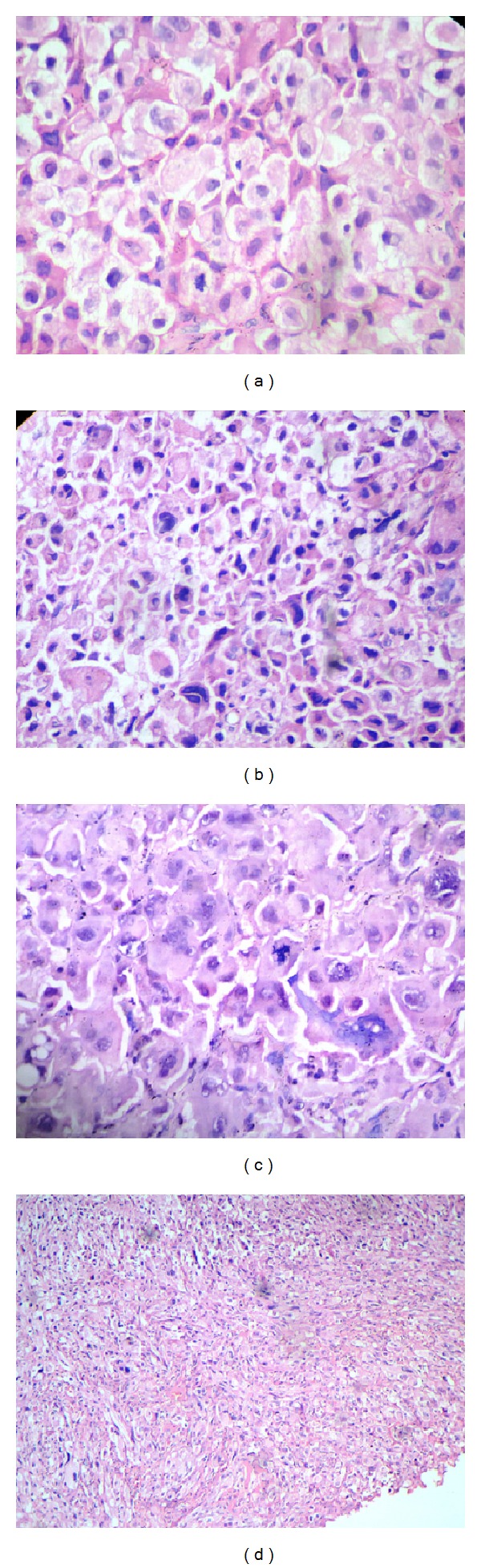
(a) Photomicrograph of amelanotic melanoma shows diffuse proliferation of large ovoid to polygonal tumor cells with pale eosinophilic granular cytoplasm and eccentrically placed nucleus (H&E 40x). (b) Nuclear pleomorphism and increased nucleocytoplasmic ratio (H&E 40x). (c) Abnormal mitotic figures (H&E 40x). (d) Tumor cells showing spindling (H&E 10x).

**Figure 3:**
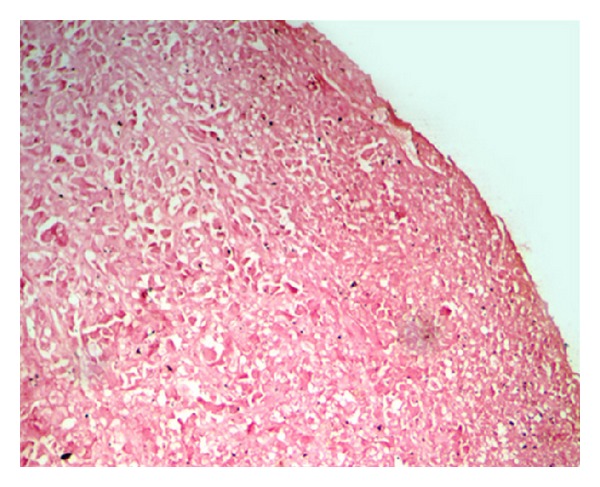
Masson-Fontana staining showing no positive cells (20x).

**Figure 4:**
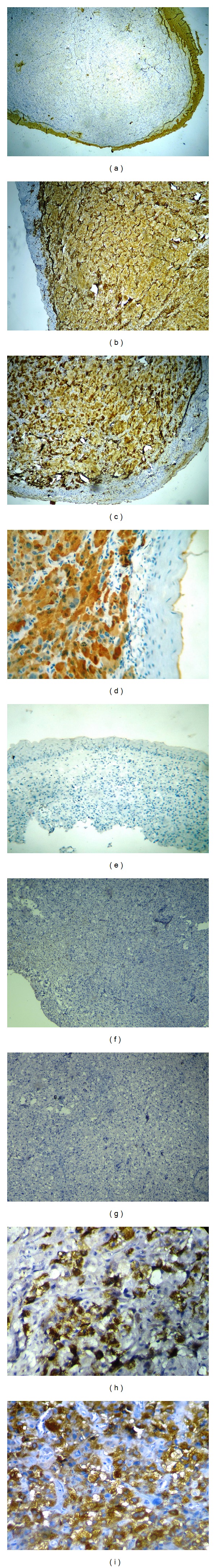
(a) Immunohistochemistry for pan cytokeratin showing no expression in tumour cells (5x). (b) Immunohistochemistry for desmin showing diffuse positivity (10x). (c) Immunohistochemistry for vimentin (diffusively positive (10x)). (d) Immunohistochemistry for S100 showing diffuse nuclear and cytoplasmic expression (40x). (e) Immunohistochemistry for CD 68 (negative, (10x)). (f) Immunohistochemistry for neuron-specific enolase (negative, (10x)). (g) Immunohistochemistry for myoglobin (negative, (5x)). (h) Immunohistochemistry showing positivity for HMB-45 (40x). (i) Immunohistochemistry for Melan-A; granular localization is observed in the cytoplasm (40x).

 Microscopically, the specimen showed sheets of large ovoid to polygonal tumor cells with pale eosinophilic granular cytoplasm and eccentrically placed nuclei in most of the cells (Figure 2(a)). Nuclear pleomorphism with increased nuclear cytoplasmic ratio was noticed (Figure 2(b)). Some cells showed vesicular nuclei with prominent nucleoli, and mitotic activity was high (Figure 2(c)). At some areas tumor cells showed spindling (Figure 2(d)). H&E staining and Masson-Fontana staining did not demonstrate melanin pigments (Figure 3). Immunohistochemically, the tumor cells were positive for vimentin, desmin, S100, HMB-45, and Melan-A and negative for cytokeratin, NSE, and CD68 (Figures 4(a)–4(i)). Based on these findings, diagnosis of amelanotic melanoma was given. The patient was referred to the Regional Cancer Center, Thiruvananthapuram. The lesion was surgically excised, and postsurgical irradiation therapy was given. 

## 3. Discussion

In the literature, amelanotic melanoma has been described under the term “the great masquerader” as clinical and histological features are often deceptive [3]. Medline search revealed 34 cases of AM in the oral cavity [4–24]. Three cases have been described in mandibular gingival area, but none of the case, has the presentation of granular cell lesion. The sites in those cases were palatal (*n* = 17), maxillary gingiva (*n* = 13), upper lip mucosa (*n* = 1), and mandibular gingiva (*n* = 3).

Based on the histological presentation, differential diagnosis included poorly differentiated squamous cell carcinoma, malignant granular cell tumor (MGCT), rhabdomyosarcoma, and malignant peripheral nerve sheath tumor (MPNST). Granular cell presentation of malignant melanoma being never reported was not included in the differential diagnosis. An initial immunohistochemical panel of pancytokeratin, vimentin, desmin, and S100 excluded tumours of epithelial origin (Figures 2(a)–2(d)) and narrowed down the possible diagnoses to MPNST, MGCT, and rhabdomyosarcoma. The next panel of immunomarkers consisted of CD68, NSE (neuron specific enolase), and myoglobin (Figures 2(e)–2(g)). However, when all of them turned out to be negative, we then considered AM, taking into consideration the S100 positivity of the tumour cells. The tumor cells were found to be positive for HMB45 and Melan-A, based on which the lesion was diagnosed as AM (Figures 2(h) and 2(i)). 

For differential diagnosis of AM, immunohistochemistry is generally used and the standard melanoma immunohistochemical antigens include S-100 protein, HMB-45, and Melan-A/MART1. Thirteen of fifteen cases of AM were positive for S-100 protein (Gibson and Goellner) [25]. In a study, Wick et al. found 92% melanomas to be positive for HMB-45 [26]. Together with Melan-A, immunostaining of HMB-45 and S100 increases the sensitivity and specificity for melanomas. Recently, microphthalmia transcription factor, tyrosinase, and Melan-A immunostains have been used to highlight melanocytes. The inclusion of these in a panel of stains for melanoma should be beneficial. Gazit and Daniels in their study showed a high immunoreactivity of oral melanoma to protein S-100 (46 cases out of 50) and HMB-45 (especially in the epithelioid variations) [27].

Several variants of AM are presently recognized, and these usually parallel the subtypes of pigmented melanoma. The more commonly known growth patterns are superficial spreading, acral lentiginous, nodular, neurotropic, and desmoplastic types/melanomas. Some unusual variants of the AM reported are adenoid/pseudopapillary, small cell, myxoid, hemangiopericytoid, and signet-ring cell melanomas [28]. In the present case, tumor cells did not reveal melanin pigment by H&E and Masson-Fontana staining. Histologically, the tumor most closely resembled the malignant granular cell tumor. Immunostaining is an essential diagnostic tool where diagnosis based merely on H&E staining becomes difficult.

Ethnicity and sun exposure appear to play a major role in the development of cutaneous melanomas while mucosal melanomas have no association with sun exposure. Cigarette smoking, alcohol consumption and denture irritation are some of the risk factors for oral melanomas [29]. The history of smoking and alcohol consumption could be possible risk factors in the present case. 

 At present, the recommended treatment is wide excision in combination with chemotherapy and, to a lesser extent, immunotherapy or irradiation therapy.

## 4. Conclusion

The diagnosis of amelanotic melanoma can be very challenging especially when they present with unusual histological appearances. Proper documentation of such cases and the role of immunohistochemistry are emphasized in this paper.
